# Social validity of work ability evaluations and official decisions within the sickness insurance system: A client perspective

**DOI:** 10.3233/WOR-213558

**Published:** 2021-09-28

**Authors:** Elin A. Karlsson, Jan L. Sandqvist, Ida Seing, Christian Ståhl

**Affiliations:** a Department of Health, Medicine and Caring Sciences, Linköping University, Linköping, Sweden; b Department of Health, Medicine and Caring Sciences, Linköping University, Norrköping, Sweden; c Department of Behavioral Sciences and Learning, Linköping University, Linköping, Sweden

**Keywords:** Acceptability, sickness absence, social insurance system, sick leave, legitimacy

## Abstract

**BACKGROUND:**

Studies of the social validity of work ability evaluations are rare, although the concept can provide valuable information about the acceptability, comprehensibility and importance of procedures.

**OBJECTIVE:**

The aim of this study was to explore clients’ perceptions of social validity of work ability evaluations and the following official decisions concerning sickness benefits within the Swedish sickness insurance system.

**METHODS:**

This was a longitudinal qualitative study based on interviews with 30 clients on sick leave, analyzed through deductive content analysis.

**RESULTS:**

Clients’ understanding of the evaluation was dependent on whether the specific tests were perceived as clearly related to the clients’ situation and what information they received. For a fair description of their work ability, clients state that the strict structure in the evaluation is not relevant to everyone.

**CONCLUSION:**

The work ability evaluations indicate low acceptability due to lack of individual adaptation, the comprehensibility varied depending on the applicability of the evaluation and information provided, while the dimension ‘importance’ indicated as higher degree of social validity. The official decision about sickness benefits however was considered unrelated to the evaluation results, lacking solid arguments and sometimes contradictory to other stakeholders’ recommendations indicating poor social validity.

## 1 Introduction

During the last decades, political reforms have been carried out in western countries to reduce rates of sick leave. The compensation for clients’ inability to work has been increasingly restricted by formalizing the criteria of access to sickness benefits [1]. Professionals in the welfare system need to express what is at stake during the decision making process, for instance eligibility for sickness benefits. In order to do so, the discrepancy between formal rules and practice needs to be bridgeable. If formal rules fail to facilitate an understanding of decisions, they will be perceived as inadequate because the decision making process is not transparent to outsiders [1]. In several studies, encounters with professionals in sickness insurance systems have had effects on clients’ self-perception, perceived social justice, motivation and ability to return to work, as their actions directly affect clients lives [2]. Assessments of work ability play a major part in facilitating authorities’ decision-making, such as upon clients’ eligibility for sickness benefits within a sickness insurance context. These assessments are crucial for clients’ to have the possibility of receiving the correct compensation for inability to work. To receive correct and fair decisions about sickness benefits it is important that the work ability assessment, serving as a foundation, is valid and reliable. There are different properties that are more or less common to investigate for instruments and procedures. Some of the more common ones are utility and content validity [3].

### 1.1 Social validity

Within vocational rehabilitation and insurance medicine, examinations of social validity are rare. Social validity was first addressed by Kazdin [4] and Wolf [5] within behavior analysis and consists of acceptability and comprehensibility of goals, procedures and outcomes, and social importance of the outcome [5–7]. Acceptability includes whether goals, procedures or outcomes are perceived as appropriate and relevant to those whom they concern. For instance, whether the content of a specific assessment is considered to be offensive or suitable. Comprehensibility includes whether the information provided to the client before, during and after an assessment is understandable and sufficient. The social importance of an assessment includes whether it was considered worth the effort in relation to what was achieved and where it led.

**Fig. 1:**
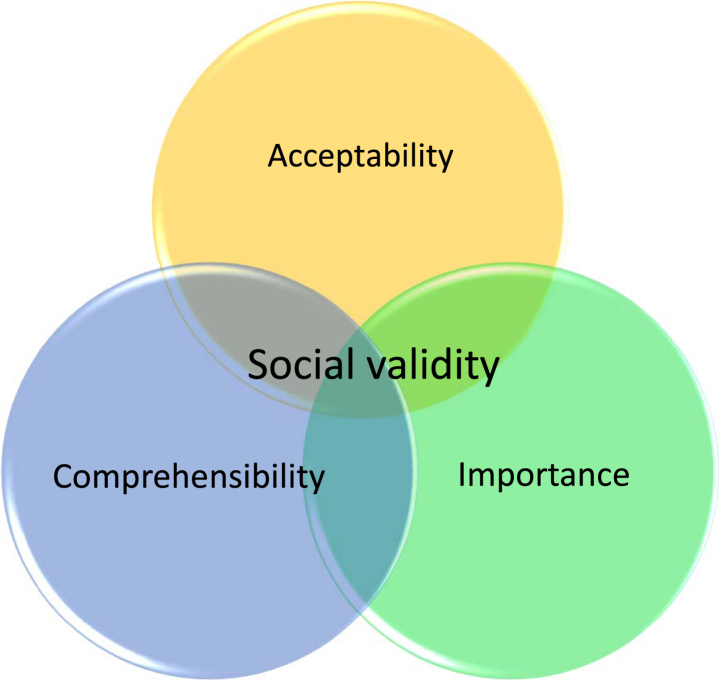
Dimensions of social validity.

Furthermore, the acceptability, comprehensibility and importance can have interactive effects. For instance, if clients receive comprehensible information about the content of a procedure and where it might lead, it is more likely that this also will enhance their acceptability and perceived importance. The concept can provide valuable information about how procedures can be improved and how stakeholders can increase clients’ motivation and participation. Socially valid procedures can facilitate clients’ motivation, participation and understanding of assessments as well as subsequent interventions and decisions [8] which make social validity interesting and relevant to investigate within the sickness insurance system. The validity of both single assessments and more comprehensive multi professional evaluations within insurance medicine are of great importance as they have a significant impact on clients’ lives both due to the economic impact of being eligible or not for sickness benefits and also regarding the interventions that these assessments might lead to. To ensure correct decisions and efforts in clients’ cases it is essential that these are based on valid results representative of how the client actually functions at work. How can correct assessment results be ensured if clients feel offended or not motivated to take active part in the assessment? Further, clients within a sickness insurance context are in a vulnerable situation where the power of balance in relation to the authorities seldom are in their favor. The encounters with the SIA and the clients’ perceived fairness of decisions may affect both their attitude towards the authority but also their possibilities to commit to a rehabilitation process, if they agree or disagree with the arguments and conclusions suggested by the SIA. This study focuses on social validity of procedures and outcomes, in terms of acceptability, comprehensibility and importance. In this case, clients’ perception of work ability evaluations within the sickness insurance system and the following official decisions.

**Fig. 2:**
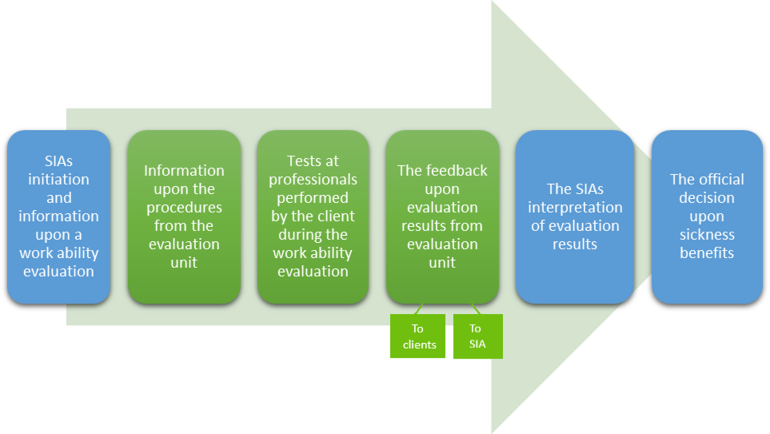
The process of work ability evaluations and official decisions shared by the SIA (blue) and evaluation units (green).

## 2 Objective

The aim of this study was to explore clients’ perceptions of social validity of work ability evaluations and the following official decisions concerning sickness benefits within the Swedish sickness insurance system.

## 3 Materials and methods

### 3.1 The Swedish sickness insurance context

The Swedish Social Insurance Agency (SIA) is a public authority with a central role in the Swedish sickness insurance system by assessing clients’ eligibility for sickness benefits and coordinating vocational rehabilitation. Workers at the SIA who decide on clients’ right to sickness benefits, will be referred to as *case managers* in this paper. In the Swedish system as in others, [1] the focus has turned to objective assessments that include professionals’ observations of their clients’ limitations, leading to the exclusion of clients’ emotional and subjective considerations. In Sweden clients are eligible for sickness benefits for up to 180 days in the sick-leave spell if they are unable to do their regular work or other work that their employer can provide [9]. The criteria for receiving sickness benefits become stricter after day 180, as eligibility is present only if they cannot perform any hypothetical work in the labor market, which corresponds with an overall increase in withdrawals of compensation after the 180-day period when there also is an increase of clients sent to work ability evaluations on behalf of the SIA [9]. Initially, a sick note from the clients’ treating physician is often enough to receive sickness benefits, but the case manager can initiate a work ability evaluation when further information is needed, where clients can either chose to participate or have their sickness benefits withdrawn. The SIAs’ work ability evaluation is an administrative tool for facilitating case managers’ decision making about clients’ eligibility for sickness benefits, performed by specific units within health care which carry out an independent assessment where the professionals involved have not previously met the client and have no role in treating the client. The professionals that can be included are physicians, where the tests can include a basic body examination and an interview, occupational therapists, who performs activity-based tests in constructed work tasks, physiotherapists, who carries out tests of physical function, and psychologists, who perform cognitive and mental tests.

When medical certificates or other documents are perceived as hard to interpret, case managers can discuss their cases with the SIA’s insurance physician, who has an advisory function within the authority. Clients who are subject to potential withdrawal of sickness benefits receive a letter with information from the SIA about the preliminary withdrawal and have two weeks to submit supplementary material to support their case, before a formal decision is concluded.

### 3.2 Study procedures

This was a qualitative longitudinal study mainly based on semi-structured interviews of clients on sick leave who had their work ability evaluated. A previous study show that clients experience of an assessment seems to be connected to whether it leads to sickness benefits or not [10], which makes it valuable to separate social validity of the work ability evaluation from social validity of the official decision, before a potential withdrawal of sickness benefits may color clients’ general opinions of the procedure. Ethical approval was granted prior to this study’s initiation (Dnr 2017/271-31). All units in Sweden performing work ability evaluations on behalf of the SIA (*n* = 35) where invited to participate in this study and those who accepted (*n* = 6) provided an information letter, to a total of 108 of their clients. A consecutive sample was conducted, with the aim of interviewing 30 clients. Written informed consent was obtained, with information about their participation being voluntary and that the researchers have no connection to the SIA and therefore are unable to affect official decisions. The first author contacted clients initially by phone, and by text or e-mail if they did not respond. Interviews were conducted in two steps: first after the evaluation but before an official decision, and the second was after an official decision had been made. The content of the interview guides was informed by social validity literature [4–8, 11–16], previous research on encounters within the sickness insurance system [9, 17, 18] and discussions with the multi-disciplinary project group. One cognitive interview was conducted with a non-academic worker to evaluate the comprehensibility of the questions which led to clarifications and a simplified language. The length of the first interviews was between 30 and 100 minutes, but mostly around 60 minutes. These interviews explored the work ability evaluation and concerned what information the clients had received before the evaluation from their case manager, if they knew the purpose with the initiation of the evaluation at the time of the performance of it and how they perceived the specific tests performed by the different professionals at the evaluation in terms of comprehensibility and acceptability. The second interviews were conducted with 27 of the original 30 participants of which one participant responded by e-mail due to difficulties in managing another oral set of questions. Three participants did not respond to the invitation for the second interview, with no given reason, despite reminders. The second set of interviews lasted for 10–40 minutes, but mostly around 20 minutes, and questions concerned consequences they experienced during and after the evaluation, what information regarding the official decision they had received from their case manager and whether they perceived the following official decision as fair and connected to the results from the evaluation. Two participants were interviewed a third time, due to events in their case being of interest, such as the SIA case manager granting sickness benefits temporarily while requesting further medical certificates. Several of the interviews were divided in shorter sessions, depending on the clients’ difficulties. The interviews took place from November 2017 to June 2018, shortly after the clients had performed a work ability evaluation (1–14 days) in most cases within the following week. In several cases clients and the first author corresponded by texts or e-mails between interviews to keep the first author updated on the case. Client files were collected from the SIA in accordance with the clients written consent, although these were sparsely used in this study due to extensive interview data thus mainly as a complement to the interviews, in order for the researchers to be able to collect information regarding, for instance, clients’ current diagnoses used in Table 1. The files will be further examined in another study. Thus, data in this study consisted of interview transcripts, texts and e-mails.

**Table 1:** Characteristics of participants

No.	Sex	Birth	Diagnostic	SA/DP/work	Duration of current sick-leave	Professionals involved in the work ability evaluation
year	category	spell at the time of first
interview, in years
1	F	1971	F, M, R	SA	3.5	Physician, Occupational Therapist, Physiotherapist, Psychologist.
2	F	1971	C	SA	6	Physician, Occupational Therapist, Physiotherapist, Psychologist.
3	M	1956	I, F	SA	4	Physician, Occupational Therapist, Physiotherapist, Psychologist.
4	M	1966	G, M	SA	6.5	Physician, Occupational Therapist, Physiotherapist, Psychologist.
5	F	1969	F, T, Q	SA	5.5	Physician, Occupational Therapist, Physiotherapist, Psychologist.
6	F	1977	F	SA &work	1	Physician, Occupational Therapist, Physiotherapist, Psychologist.
7	M	1954	T, H	SA	2.5	Physician, Occupational Therapist, Physiotherapist, Psychologist.
8	F	1965	L	SA	2	Physician, Occupational Therapist, Physiotherapist, Psychologist.
9	M	1980	F	SA	2	Physician, Occupational Therapist, Physiotherapist, Psychologist.
10	M	1970	M	SA	2	Physician, Occupational Therapist, Physiotherapist, Psychologist.
11	M	1970	F	SA	2.5	Physician, Occupational Therapist, Physiotherapist, Psychologist.
12	M	1982	R, M, T	SA	4	Physician, Occupational Therapist, Physiotherapist, Psychologist.
13	F	1965	F, R	SA	16.5	Physician, Occupational Therapist, Physiotherapist, Psychologist.
14	F	1959	M, F	SA	0.3	Physician, Occupational Therapist, Physiotherapist, Psychologist.
15	F	1980	F	SA	2.5	Physician, Occupational Therapist, Physiotherapist, Psychologist.
16	F	1961	M, F	SA	1	Physician, Occupational Therapist, Physiotherapist, Psychologist.
17	M	1976	M	SA	6	Physician, Occupational Therapist, Physiotherapist, Psychologist.
18	M	1962	M	SA	1.5	Physician, Occupational Therapist, Physiotherapist, Psychologist.
19	F	1981	F	SA	3	Physician, Occupational Therapist, Physiotherapist, Psychologist.
20	F	1965	M, G, F	SA	9	Physician, Occupational Therapist, Physiotherapist, Psychologist.
21	F	1980	F	SA	3	Physician, Occupational Therapist, Physiotherapist, Psychologist.
22	F	1956	M, F, T	SA	2	Physician, Occupational Therapist, Physiotherapist, Psychologist.
23	M	1956	M	SA	1.5	Physician, Occupational Therapist, Physiotherapist, Psychologist.
24	F	1967	I, M, J	SA	0.5	Physician, Occupational Therapist, Physiotherapist, Psychologist.
25	F	1963	M, F	SA &work	2	Physician, Occupational Therapist, Physiotherapist, Psychologist.
26	F	1956	F	SA	1.5	Physician, Occupational Therapist, Physiotherapist, Psychologist.
27	F	1957	I	SA &work	4	Physician, Occupational Therapist, Physiotherapist, Psychologist.
28	F	1970	M, T	SA &work	23	Physician, Occupational Therapist, Physiotherapist, Psychologist.
29	M	1984	F, L, K	SA	4	Physician
30	M	1960	G, M	SA	2	Physician, Occupational Therapist, Physiotherapist, Psychologist.

SA/DP/work refers to sickness absence (SA), disability pension (DP) and part time work. Diagnostic categories refer to the diagnostic manual ICD-10: Mental and behavioural disorders (F), Diseases of the musculoskeletal system and connective tissue (M), Symptoms, signs and abnormal clinical and laboratory findings, not elsewhere classified (R), Neoplasms (C), Diseases of the circulatory system (I), Diseases of the nervous system (G), Injury, poisoning and certain other consequences of external causes (T), Congenital malformations, deformations and chromosomal abnormalities (Q), Diseases of the eye and adnexa/Diseases of the ear and mastoid process (H), Diseases of the skin and subcutaneous tissue (L), Diseases of the respiratory system (J), Diseases of the digestive system (K).

### 3.3 Participants

The participants consisted of 12 males and 18 females, aged 36–64 years, representing several geographic parts of Sweden. The majority of the participants were on sickness absence full-time, but a few also worked part time. Their difficulties represent a variety of diagnoses, often mental and musculoskeletal disorders and often combinations of several different diagnoses. All participants except one, had seen a physician, occupational therapist, psychologist and physiotherapist during the work ability evaluation (see Table 1).

### 3.4 Data analysis

A qualitative deductive content analysis [19] was used for the interviews, texts and e-mails and started when all interviews were performed. Categories and subcategories were specified based on aspects of interest to evaluate from the social validity literature [4–8, 12–15] and this study’s interview guide. The work ability evaluation and the following official decision were intentionally separated into different categories in the analysis in accordance with this study’s aim and design. Data were first skimmed, then read more carefully as meaning units were categorized. The data were read through once more to ensure that no meaning units of relevance were left out. This was done until the chosen meaning units and their categorization seemed concordant. As customary within deductive content analysis, the process moved from deductive to inductive back and forth as categories were revised and new subcategories were identified. The first author performed these steps; however, these were discussed together with the second author and the fourth author. All authors have experience of qualitative research.

## 4 Results

The social validity of work ability evaluations and official decisions will be presented in terms of acceptability, comprehensibility and importance (see Table 2).

**Table 2:** Dimensions of social validity related to the work ability evaluation and/or the official decision

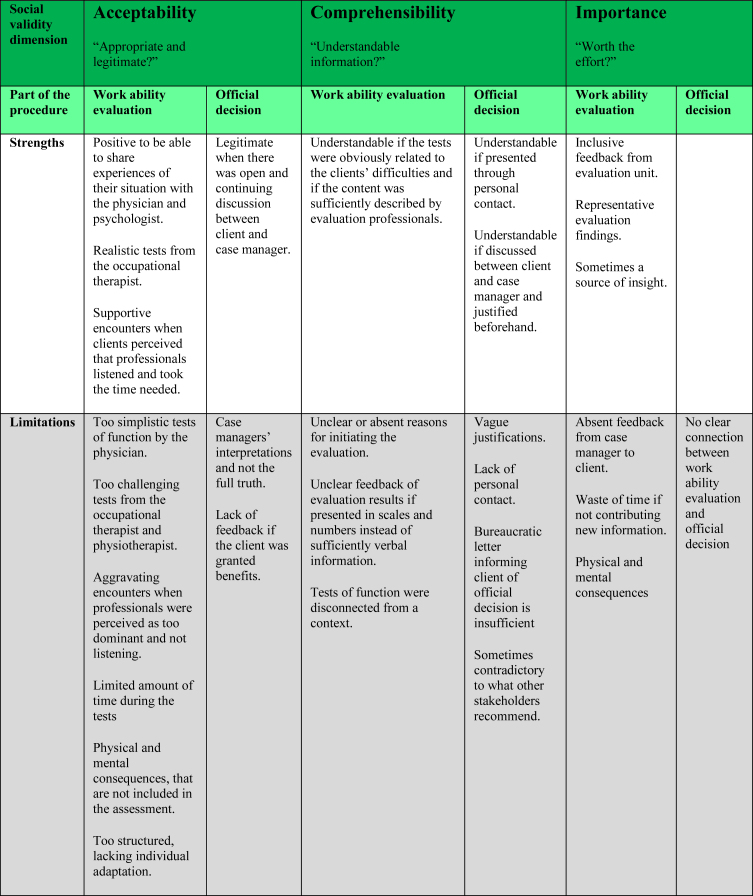

### 4.1 The work ability evaluation

#### 4.1.1 Acceptability of the work ability evaluation

*4.1.1.1 The perceived legitimacy of professionals’ tests:*Clients described hesitation regarding the physicians’ tests, due to perceiving them as consisting mainly of going through previous documentation or a too simplistic examination of functions.

*What he [the physician] did, I had to take off my clothes, and he pushed, you know, pushed [my back] more or less, it was so basic, as okay you have marked that you have pain in your back on the right side so then I push some right here. No further comments, I just push there. So absurd because it has nothing to do with the matter*” [Client 5].

However, clients also stated that they appreciated the opportunity to provide the physician with information about their background which was also expressed after seeing the psychologist, as in clients appreciating someone talking to them and asking them how they feel. Regarding the occupational therapist, clients describe performing tasks similar to what they would do at a workplace. Clients seemed to accept these tests due to their resemblance to work and due to the occurrence of realistic distractions.

“*That test at the occupational therapist was very much what I struggle with at work many times, that people are coming in and it pops up e-mails and it tings, and you get interrupted. That is just what tires me many times, because I have such a hard time maintaining concentration then*. . . *And it felt like it was that situation, they managed to get about the same environment, so it made, it felt like the difficulties were obvious then. So, in that sense it felt good*” [Client 27]. 

However, some clients perceived these tasks as too demanding and ruthless, for instance those with difficulties that arrived days after the test, such as pain. These opinions also exist in relation to the physiotherapists’ tests, where clients expressed that they were too challenging.

*4.1.1.2 The perceived generalizability and fairness of the tests:*The evaluation results are in the majority of cases described as representative of how the client’s function, although clients also state that important parts have been left out, such as other diagnoses they have or the consequences after performing activities in their daily living. In some cases, clients state that they were worse off than expected, describing the evaluation as a source of insight. Several clients who previously were skeptical of the tests were surprised when their difficulties were noticed and described in a fair way, especially due to the limited amount of time in the evaluation. Time is a major concern raised by the clients, stating that, depending on their difficulties they can perform quite well during a limited amount of time, but the consequences afterwards are not taken into consideration which made clients question the legitimacy of the evaluation.

“*I think it’s a very shallow evaluation, it doesn’t say for example how a person would respond if they work with that task a whole day*. . . *it just gives a simple direction, can he lift a certain thing up and down. Well, I don’t know if that is relevant*” [Client 9].

The appropriateness of the evaluation is perceived as depending on how tests are adapted to the client’s difficulties. Clients state that tests need to be individually adapted to each client’s prerequisites. For instance, if the client’s function is at best in the morning, the evaluation should also be performed in the afternoon, in order to give a fair description of the client’s work ability. Clients also describe preferring that professionals were more thoroughly informed about their difficulties and needs, in order for them to test what is relevant and appropriate, not forcing all clients to perform the exact same tests independent of the cause of sickness absence. Clients described understanding the general controlled procedure of the evaluation and that its included tests must be able to capture work ability of a broad variety of clients. However, clients also highlighted that the structure is too strict, neglecting nuances and explanations in the evaluation results that do not fit into the forms.

*4.1.1.3 Encounters during the work ability evaluation:*The encounters with professionals during the evaluation affected clients’ perceived acceptability of the evaluation. Positive encounters are described, including professionals introducing themselves properly, listening to the client and providing professional treatment focusing on the client. Other aspects of experiencing the encounters as positive were a calm approach, as in professionals taking their time to assess the client, showing commitment, communicating in a respectful way and trying to make the clients feel comfortable.

“*It is often enough that they just ask you how you are and how you feel and that* –*it is so tremendously simple to ask a thing like that and instantly it feels a lot better*” [Client 11].

Aspects aggravating the encounters were when professionals were perceived as absent by not paying attention to what the client said, or when the professional was perceived as too dominant or arrogant. There were descriptions of encounters where professionals seemed mentally and/or emotionally absent and clients reported that this caused them frustration and an even more vulnerable position as they did not feel the professional was listening to them. Furthermore, there are examples of clients reporting that they struggled during the tests and felt neglected due to professionals encouraging them to carry on with the next test without a pause and without questions about how they felt. Some clients described frustration when the professional appeared to focus only on the tests and not the actual person in front of them.

“*They don’t take you seriously but tear and pull at the legs, as if they weren’t, as if they were butter. You say that you are in pain,* ‘*yes but you have full mobility’, well but it hurts like hell once you strain it*” [Client 17]

#### 4.1.2 Comprehensibility of the work ability evaluation

*4.1.2.1 The initiation of a work ability evaluation:*According to the interviewed clients, the purpose of the evaluation was not always expressed clearly by the case manager at the SIA, although clients sometimes had an idea of why the request occurs, such as unclear medical certificates, being sickness absent for ‘too long’, their difficulties being questioned, or the case manager considering disability pension. Clients were notified by the case manager that the evaluation would take place in different ways, such as by phone or in a personal meeting, or just by a letter without any oral explanation, and in rare cases through discussions during status-meetings with different stakeholders. Hence, some clients stated they had received a clear explanation about the purpose of the evaluation but the majority in this study had not.

“*It was just some woman [from the SIA] calling me, saying that* ‘*we will send you on an evaluation and you will receive a letter from the evaluation unit*’. . .*And then I did, mm.*

***Did you receive an explanation about why, and what the evaluation will be used for?***

*No. No, nothing, she just called and said* ‘*we are sending you on an evaluation*’ ” [Client 22]. 

Furthermore, clients expressed their sense of powerlessness in having no authority to refuse to participate out of fear of having their sickness benefits withdrawn. There are also several examples when case managers have told the clients that the evaluation is costly and that they should be grateful.

“*Then he [the case manager] says to me* ‘*Just so you know, we are spending a lot of money on you now, so are you going to do this properly?*’. . .*There are no options, to me I just have to consent to being in their possession. I have nothing to say in this, because then I will lose my money and what am I then going to live on?*” [Client 21]

Clients stated they received a letter with information from the evaluation unit beforehand. Several stated that the information was sufficient, but several would have preferred details about the content and what kinds of tests would be included beforehand, to be able to prepare themselves and to reduce anxiety. Clients described feeling anxious beforehand, but there are also positive expectations such as hoping to receive thorough documentation and hoping for a positive change in their situation. Some questioned the initiation due to existing extensive documentation of their difficulties from other stakeholders, and how another evaluation would contribute.

*4.1.2.2 Comprehensibility of the tests included:*Clients confidence in the different tests and the comprehensibility of them were dependent on whether the specific tests were perceived as clearly related to the clients’ difficulties and whether the content and purpose was sufficiently described to the client by the evaluation professionals. For instance, clients with physical difficulties often questioned why they had to see a psychologist, and clients with depression questioned why they had to see the physiotherapist. The psychologists’ tests could include memory tests like repeating numbers back and forth, which was comprehensible to some but several of the clients could not see a clear connection to their work ability. The tests at the physiotherapist could consist of walking up stairs on time and drawing a circle with stretched arms. These tests were in general perceived as comprehensible in terms of testing physical function, although the tests were also criticized for being disconnected from a context.

“*The tests you have to expose yourself to are sick, it feels humiliating to stand there and to be evaluated*. . . *You have made a circle in a minute with left hand, then you can work*” [Client 1].

*4.1.2.3 The comprehensibility of the provided evaluation results:*Clients’ stated that they received feedback upon the results of the work ability evaluation from the physician at the evaluation unit on the last day on the evaluation or the week after, either in person or by telephone. This feedback was considered understandable and appreciated when the results were explained and justified in a concrete way and when clients were given time to discuss the findings, but too general or difficult to comprehend when presented in scales and numbers. In some cases, a further evaluation by the treating physician was recommended, for instance when a completely new diagnosis was considered. This sometimes left the client with questions that were not answered.

“*I was told he thought, the psychologist thought that there were things indicating ADHD and he recommended an evaluation. And then I felt like this that well, I would have appreciated discussing this with the psychologist since I have seen psychologists for so many years and I have met many professionals and none of them have ever mentioned this at all. So I would have liked to question that at the psychologist, what makes you conclude this*” [Client 28].

#### 4.1.3 Importance of the work ability evaluation

*4.1.3.1 Lack of feedback from the SIA case manager:*Clients stated that they did not receive any feedback from the case manager after the evaluation apart from the official decision that often arrived by letter several months later. In rare cases the case manager called the clients after the evaluation to let them know that documentation from the evaluation had arrived and to ask the client how he/she feels, which was much appreciated. Clients also described taking the initiative to contact their case manager with questions about where it would lead, but that preferably the case managers should be the ones initiating contact to explain their interpretation of the documentation. This lack of response from the case manager made clients question the importance of the evaluation and how it was used in their case.

*4.1.3.2 The work ability evaluations contribution to the client’s case:*Clients perceived the evaluation mainly as a way of confirming what their treating health care professionals already knew. Clients highlighted the lack of cooperation between the SIA and other stakeholders, for instance when the client previously had been on a similar evaluation, which showed the same results as this one, they often perceived this work ability evaluation as a waste of time.

“*Well, my treating physician knows all these things already, she has known for a long time, but for the SIA I think it was very helpful, because they, they have a very hard time understanding your situation. So, for them I think this was quite important. But regarding my treating physician who I have had for several years, it’s like, well this is what she has been trying to explain the whole time, when she has received e-mails from the SIA*” [Client 2].

Clients also appreciated receiving up-to-date documentation, especially in relation to their contact with the SIA as they found it useful to have a new document proving their limitations. There were cases where clients’ diagnoses were changed which could be considered both helpful and provoking and cases where the evaluation contributed details that facilitated clients own understanding of their difficulties.

“*I found out things that I didn’t know about myself, both mentally and physically, so to me this was a really giving journey and I realized that my body, I live like in a mendacious world, if I say so. I think that I am better off than I am*” [Client 10].

*4.1.3.3 Consequences for the clients physical and mental well-being:*During the evaluation clients stated they became physically exhausted and in pain, and some described the evaluation as mental and physical abuse. Clients highlighted the delayed consequences such as pain and fatigue leading to limitations such as being bed-bound, for days or weeks after the evaluation. Clients described having to deal with emotions afterwards such as feeling depressed and powerless, and that they were left with these emotions alone when the evaluation ended. Clients also described being mentally exhausted feeling distrusted by the SIA as their own descriptions were diminished due to someone else judging how restricting their difficulties were, or feeling bad when they did not manage the tests in the way that they had hoped.

“*You have some free time in between which is fortunate because you, you cry and sob like a fool all the time*. . . *the sum of it all was that I felt completely worthless*” [Client 6].

The majority of the clients who experienced these consequences are critical of the evaluation. Some described that they accepted these consequences as a way of receiving proper documentation that will satisfy the SIA case manager, or because ‘this is the same for everyone on sick-leave’, or because this is a normal reaction to them performing these activities hence expected. Clients described how their everyday lives were affected, in terms of household activities being impossible to manage or not being able to help their children with schoolwork, due to all energy spent on the tests during the evaluation. A few clients reported that they were not affected physically or mentally by the evaluation. Clients also highlighted associated aspects of the evaluation, such as the travel to get there. The distance to the evaluation unit could be 300 kilometers one-way, leading to difficulties regarding travel. For instance, some had arranged for a ride home, but several of the clients were not prepared on how they would be affected by the evaluation and thus struggled to get home. Examples highlighted were being forced to make multiple stops on the way home by car on the highway due to exhaustion. Clients stated they would have appreciated being more prepared about how exhausting the evaluation may be.

### 4.2 The following official decision about sickness benefits

#### 4.2.1 Acceptability of the official decision

*4.2.1.1 The lack of personal contact:*Several months after the work ability evaluation there were still several clients who had not received any decision or feedback from the SIA. Although the medical certificates and payments of sickness benefits may have continued as usual, clients stated they would prefer to be kept updated on the process by receiving feedback also if granted continued sickness benefits. By not receiving feedback they were insecure about whether the case manager had made a decision in their case and were often hesitant to make contact, hence thinking it would be better to ‘let sleeping dogs lie’. Clients stated that a decision from the SIA of withdrawn benefits preferably should be carried out through some kind of personal contact, for instance by telephone or a meeting. There are descriptions where clients perceived that their case manager deliberately avoided personal contact with them regarding the decision, including when the clients were speaking to them about other matters.

“*I spoke to her [the case manager] the day before, since I wanted financial compensation for the travels to the evaluation unit. And then, then I asked her at the same time if anything new had happened in my case, and then she said* ‘*well it’s not sure it’s enough*’ *[to receive sickness benefits]. And the day after I had those documents here [a preliminary decision of withdrawal of sickness benefits, by letter]*. . . *she was- it was (laughter)*. . .*she was on her way to finishing the phone call when I just sort of blurted* ‘*well how are things going with the other stuff? Have you done anything, is something coming up?*’ *So she really had not planned to say anything, she hadn’t*” [Client 14].

*4.2.1.2 The perceived fairness and predictability of the decision:*In terms of fairness, clients highlighted that the conclusions made by the case manager were interpretations and not the full truth. In some cases, clients stated that it worked out well in the end, but not due to a just decision but rather to their own ability to adjust to the situation. Clients also highlighted that their expectations beforehand were low due to negative media attention against the SIA and what they have heard from others about the contact with the authority. With this in mind, some clients experienced an unfair decision as predictable, due to low expectations. What clients perceived as fair differed from one client to another, mainly depending on the clients’ situation and what arguments and information were given. For instance, clients who were recommended to receive disability pension could either perceive this as fair, if they agreed with the arguments and conclusions, or unfair if they felt that they were judged unable to work too soon and not given a proper chance to return to work. Those clients who generally perceived the official decision as fair are those who had a clear idea from the initiation of the work ability evaluation regarding where it might lead and who had an open discussion with their case manager, either if it led to disability pension or withdrawn sickness benefits. Some clients also stated that they believed the case manager had already decided on their eligibility for sickness benefits before the work ability evaluation and chose to interpret the parts of the evaluation that supported their opinion.

“*They [the SIA], as far as I understood, they want closure, that’s what it’s about*. . . *Because I, it feels, it doesn’t feel like- Well, it’s been an uphill all my life, so I don’t know, I won’t get that closure, but they will, so I guess that’s good for them*. . . *I still have to climb that damn hill*” [Client 29].

#### 4.2.2 Comprehensibility of the official decision

*4.2.2.1 The case managers’ justification of an official decision:*Before receiving a letter of preliminary withdrawn sickness benefits a few clients stated that they received a telephone call preparing them or a status meeting where possible alternatives were discussed with other involved stakeholders. Those receiving feedback also through status meetings (and not only by letter) were generally more positive and seemed to understand the decision quite well. Bureaucratic letters are perceived as insufficient and not comprehensible if the content is not explained with solid examples, although it was common that clients received only the letter. Those clients who perceived the information and decision from the SIA as being least comprehensible are those for whom the decision was contradictory to their treating physicians’, other professionals’ or other stakeholders’ recommendations or when the clients perceived that their situation and work ability had not changed, although the case manager may have decided that they suddenly are able to work. The shift from assessing clients’ specific work ability to assessing their general work ability from day 181 in the sick leave spell was not perceived as comprehensible or legitimate. Clients seemed to understand their options, for instance that they must initiate work training in order to receive benefits, but the reasons are not always understood. Vague motivations from case managers referring to unspecific jobs that only potentially would work for the client is one aspect highlighted by the clients in terms of lacking comprehensibility.

“ ‘*The SIAs assessment is that your work ability is not reduced by a quarter, due to illness or injury. In relation to a normally occurring work that is physically light, not cognitively demanding where you can vary position and do not need to walk much*’. . . *and I imagine that they leave it up to the Public Employment Office to figure out*” [Client 24].

Clients described their sense of powerlessness both in relation to the case manager but also in relation to the insurance physician and expressed they sometimes behaved passively due to feeling that they had no say or not enough energy to ask for clarifications. Those who perceived the decision from the SIA as most comprehensible were those cases where it had been discussed beforehand and the clients had been given time to prepare themselves. For instance, in cases where disability pension had been expressed as the purpose when initiating the evaluation, perhaps also in line with recommendations from the treating physician, or when a forewarning had been given through personal contact with the case manager before a letter of preliminary withdrawal of sickness benefits entered their mailbox.

#### 4.2.3 Importance of the official decision

*4.2.3.1 The connection between the work ability evaluation and the official decision:*The majority of the clients had the same opinion of the evaluation both before and after an official decision. However, there are clients whose opinions of the work ability evaluation were colored by the official decision, for instance when clients were critical of the evaluation but afterwards could appreciate it through feedback from their case manager upon a decision about granted sickness benefits. Another example was when clients appreciated the evaluation and recognized the results but felt fooled when the case manager withdrew their benefits despite other recommendations from the evaluation unit which also affected their opinion in terms of perceiving the evaluation as a false show. Clients described an unclear connection between work ability evaluation and the following decision from the SIA, in fact there was a perceived discrepancy between the conclusions from the evaluation unit and the official decision about sickness benefits. Clients stated that the findings from the evaluation unit were representative but that the decision from the SIA was not.

“*That’s where the double violation is, the SIA run over all the experience of my treating brain rehabilitation professionals, so this doesn’t count. Then the same thing with this institution [evaluation unit], that the SIA buy a service off, so that the only, the only voices outweighing this is this case managers interpretation and the SIAs own insurance physician. Two people who have never met me*” [Client 5].

Clients also stated that the evaluation was important for their case, providing a foundation for the continuation, such as disability pension, rehabilitation, or return to work, although they felt the evaluations were important mainly when being granted further benefits. However, these cases are rare compared to those perceiving the decision as being completely unrelated to the evaluation results.

## 5 Discussion

This study explored clients’ perceptions of the social validity of work ability evaluations and the following official decisions concerning sickness benefits within the sickness insurance system. Carter [11] argues that just because a program has shown to be effective it does not mean that it is appropriate and socially valid for those involved. When the understanding of a procedure is insufficient the result is low compliance and low importance. Therefore there is a need to make sure that sufficient information is given as a part of enhancing acceptability [11], although the assessment itself needs to include relevant and appropriate tests in order for the dimension ‘acceptability’ to be considered socially valid. This should be even more important in intrusive procedures, i.e. those thought to cause physical or psychological distress [20] such as work ability evaluations and decisions within a sickness insurance context.

As shown in Table 2, the dimension ‘acceptability’ is affected by an extensive number of factors, some of which limit the acceptability of work ability evaluations, for instance that the consequences clients suffer after the tests are not taken into consideration in the evaluation but only the results on the tests. In another study [21] physicians stressed the need for several hours of rest or sleep after a work day as a very strong reason for certifying sickness absence, which is in line with the holistic view upon work ability in terms of sustainability as opposed to what the SIA’s work ability evaluation includes [22]. The SIA assesses a reductionistic version of work ability [22] using a biomedical perspective focusing rather on function compared to other more holistic approaches in assessing work ability from a biopsychosocial or ecological perspective [23] which may be used by other stakeholders in the welfare system. The clients described how this reductionistic view upon their abilities and difficulties made them question the legitimacy and acceptability of the evaluation. Furthermore, the standardized structure is described both as appropriate, since the tests must capture work ability for a broad variety of clients, but also as inappropriate by being too strict neglecting nuances and explanations that do not fit into the forms. Clients stated that the tests need to be individually adapted. However, the SIA aims to receive an independent assessment performed equally regardless of the cause of sickness absence. There are studies [24] stating that treating everyone the same and assuming this is fair simply does not work. Public organizations based on rationalization understood as standardization will have difficulty responding to diverse citizens’ needs and prerequisites, leading to citizens feeling unfairly treated [24]. Sometimes we need to treat everyone the same, but at other times we need to treat people differently in order to provide fairness, which leads to the following question: “When should we treat people differently to be fair and when must we treat them the same?” [24]. There has been a movement in many western countries towards more objective and standardized methods for assessing work ability [1]. Finding an appropriate balance between standardization and flexibility is a challenge. Although the SIA’s goal of equitable and just assessments is appropriate, the procedures in achieving this may not be appropriate for all clients due to the lack of flexibility. Without the possibility of adapting the evaluation to specific clients’ needs and prerequisites, the provided information to clients becomes even more important, leaving case managers and evaluation professionals with a challenging task in justifying procedures to enhance the social validity. Aspects of enhancing acceptability of the work ability evaluations are supportive encounters when professionals are perceived as good listeners, as has been found in other studies [17] and when professionals take the time needed during the tests. Furthermore, an open and continuing discussion between client and case manager through the process also facilitated clients’ acceptability. Clients seemed to accept the tests performed by occupational therapists due to their resemblance to work, and clients seemed to comprehend the physiotherapists’ tests in terms of testing functions. But these tests are also considered ruthless and too demanding. In summary, there seems to exist poor social validity in terms of acceptability for the work ability evaluation as a whole, although there are parts that are more acceptable than others.

This study shows that the information provided to clients within the dimension ‘comprehensibility’, has a key role in clients perceptions of following steps in the process. Some clients received a clear explanation from their case manager about the purpose of initiating a work ability evaluation but the majority in this study had not. This finding is similar to another study [25] where the majority of clients were simply notified by the case manager that an evaluation would take place, but in rare cases this was discussed at a status meeting with the client and other stakeholders as an option which all agreed upon. Clients stated they would have preferred a variety of more information beforehand, both regarding the content of the evaluation and to hear solid arguments justifying the initiation of the evaluation, especially when existing documentation in the clients’ case was already extensive. This suggests that the information case managers provide to client’s needs to be individually adapted and that client’s need to have the possibility to get in contact easily with their case manager when questions emerge. Authorities need to express and justify the reasons for a diverse range of steps during clients’ sick-leave process, not only regarding official decisions. Also another study [26] highlight the importance of a fair, open, and respectful communications from system representatives, and the provision of clear and thorough information to clients, in order to prevent the claim process from having a negative impact upon clients mental health. In another study [17] case managers perceived the work ability evaluations as a means towards transparency which was thought to facilitate the clients understanding and acceptance of the official decision, due to case managers having to discuss the initiation of an evaluation, what it will be used for and the potential outcomes. The case managers perceptions in this study [17] mirror what the interviewed clients in the present study state they would want but what few report they had received. By facilitating clients’ understanding of the different steps in the sick-leave process, this will most likely increase the comprehensibility of the work ability evaluation and the subsequent official decision. There seems to exist a varying degree of social validity in terms of comprehensibility for the evaluation that is dependent on whether or not the tests are applicable to the clients’ situation, and what information the client has received. However, there is only so much a proper explanation can do for increasing social validity as a whole, as the dimensions of acceptability and importance are just as important as comprehensibility to manage.

Regarding the third dimension, ‘importance’ for the work ability evaluation, the results indicate a more positive perception since the evaluation in general was considered important and worth the effort. Reasons for this were due to clients’ perceiving evaluation findings representative for how they function and due to inclusive feedback from the evaluation units where they were given the opportunity to discuss and correct the findings. The evaluation was considered to be particularly important if it also contributed new information which is concordant with another study [17]. However, the consequences of participating in the evaluation were described in terms of exhaustion mentally and physically, similar to another study [17] and included pain and an inability to perform activities of daily living which could last for days or weeks. These consequences made clients question whether the evaluation was worth the effort and whether this really was the only way to verify their abilities and difficulties. The work ability evaluation can be considered socially valid in terms of importance, although the potential consequences should be considered to a higher extent. For instance, by discussions and preparing the clients before the initiation of the evaluation, as suggested by this study’s clients.

The official decision about sickness benefits on the other hand, was considered to be unrelated to the evaluation results, a matter of case-managers interpretation, lacking solid arguments from the case manager, and sometimes contradictory regarding other stakeholders’ recommendations, which indicates poor social validity in terms of acceptability, comprehensibility and importance. Furthermore, clients raised the issue of absence of feedback about official decisions when granted sickness benefits, similar to another study [17], which indicates that the work ability evaluation is seen mainly as an administrative tool by the case managers who have no legal obligations to make contact unless a withdrawal is considered; although a communicated decision also when benefits were granted could reduce the duration of clients’ anxiety and uncertainty. Cooper [24] states that a basis for trust in public organizations is transparency. The SIAs focus has moved from working on enhancing citizens trust to a focus on legal security which has resulted in an increase of standardized methods and tools [27]. Standardized methods may have high reliability but as the control of procedures enhances, the validity and utility often decrease [28, 29] which may be a reasonable explanation for this study’s results as the SIAs exercise of authority and current regulations limit possibilities for individual adaptations and the inclusion of subjective information. Clients may or may not understand (or agree with) the legislation and policies that Social Insurance Agencies are bound to follow, which may be obvious and logical from a case manager perspective. Clients’ frustration regarding SIA regulations as well as difficulties in understanding the sickness insurance system have been highlighted in other studies [25]. A study [25] based on client files identified a discrepancy in how work ability evaluations are interpreted as clients in their argumentation for their case referred to quotes from the documented work ability evaluation that supported being eligible for sickness benefits, while the case manager referred to other parts of the evaluation supporting a decision to withdraw them. Also another study [17] describes the interpretations of evaluation results as problematic, as clients considered the assessment to be fair, but the interpretation of it, and hence the decision, as unfair. If formal rules fail to translate into a reasonable and fair practice, the methods used, in this case particularly official decisions, are unlikely to reach social validity from a client perspective. The SIA’s current exercise of authority in accordance with regulations and policy may not be compatible with socially valid official decisions. Potential options to enhance social validity for the official decisions are that the SIA and their case managers keep their communication open and continuous with the clients, which in this study was a factor that enhanced clients’ perceptions of the official decision as legitimate and acceptable, although this is just one piece of the puzzle. This is in line with another study [17] where feedback and a dialogue between client and case manager increased acceptability. Furthermore, the feedback about an official decision in this study was considered to be more comprehensible if presented through personal contact, i.e. phone or a meeting. These aspects concerning personal continual contact raised by the clients in this study are examples of efforts that have been reduced by the SIA in the previous years after the shift in focus from trust to legal security [17], and which may aggravate one of the SIAs previous goals: to enhance clients’ understanding of official decisions.

### 5.1 Methodological considerations

This study’s trustworthiness will be discussed in terms of transferability and credibility according to Patton [30]. A strength regarding credibility was the design of interviews in two steps as earlier studies have found that the experience of an assessment seems to be connected with whether it leads to sickness benefits or not [10]. By performing interviews in two steps, the described experiences of the work ability evaluation would not be colored by a potential official decision of withdrawn sickness benefits. However, the dependence of the outcome that was shown in the previous study [17] was not prominent in this one. Both smaller and larger evaluation units were included which is a strength that increases this study’s transferability, representing different types of evaluation units. The flexibility when conducting interviews, by dividing one interview into two or three occasions or allowing one participant to answer questions also by e-mail, could be considered as a limitation since the procedure has to some extent been inconsistent. But the argument for keeping a flexible structure in this way is the fact that this facilitates participation for a diverse range of clients which increases transferability. In most cases the first interview, which was the most extensive one, was conducted within the following week after the work ability evaluation which counteracted potential recall bias. To strengthen credibility there were several qualitative researchers involved in the analysis process, for example by discussing content and categorization. All of the authors have performed qualitative studies before. The documents collected in this study were only partially used, in order to facilitate the researchers understanding of the process in the clients’ cases and to gain additional demographic information. These documents will be more thoroughly analyzed in another study. For data analysis, a deductive content analysis was used [19], which was considered to be appropriate as this was an evaluation of social validity.

Evaluations of social validity is unusual within vocational rehabilitation and insurance medicine, leading to theoretical inspiration being found in other fields, such as behavioral analysis. How applicable the content of the concept is to a sickness insurance context can be discussed as a potential limitation. This process of transferring potentially relevant aspects from one field to another needs to be further explored which may be of interest in future studies. In this study, the concept was useful by providing inspiration and a framework on what aspects could be included. By exploring previous research and theory on the concept, it became clear that there is no consensus on what aspects that are the most important ones or even which ones should be included. However, there were clear patterns in other studies and theoretical texts upon the concept, such as relating the consequences of a procedure to social importance and relating whether a procedure was perceived as appropriate or insulting to acceptability. The literature describes the importance of comprehensible information, but this is given sparsely room within research where the main focus is on studying acceptability and importance. Within a sickness insurance context, information about processes in the system is essential and the delivery of comprehensible information one of the difficulties in a bureaucratic organization such as the SIA. Therefore, comprehensibility has been given more room in this study’s conceptualization of the concept, as a contextual adaptation. This showed to be quite useful in this study since these finding to some extent could explain how the provided information affected clients experience of the process, but it also made the analysis more complex since there was a clear interactive effect between acceptability and comprehensibility. In relation to current regulations in the Swedish sickness insurance system, there were some difficulties in applying the concept which is also clear in the results. The official decision showed to be socially invalid in terms of acceptability, comprehensibility and importance, which makes one question whether it is actually the regulations that are not compatible with socially valid official decisions rather than the decisions in itself. Work ability is as previously mentioned assessed by using a reductionistic approach by the SIA excluding factors that clients and other stakeholders often include when talking about and assessing work ability, i.e. a holistic approach as described by Ståhl et al. (22). This can be confusing and frustrating for clients, who in this study perceived that their case managers made far-fetched interpretations of assessment results justifying these with vague arguments, or when other stakeholders’ recommendations and SIA decisions counteract. This affected the acceptability, comprehensibility and the importance of the official decisions as well as some parts of the work ability evaluation.

## 6 Conclusion

Social validity of work ability evaluations and official decisions was explored by using three dimensions of the concept: acceptability, comprehensibility and importance, leading to some parts being socially valid while others were not. There seem to exist poor social validity in terms of acceptability of work ability evaluations within the sickness insurance system, due to the lack of individual adaptation and neglecting consequences after the tests. In terms of comprehensibility, work ability evaluations are comprehensible depending on the applicability of the clients’ situation and the provided information from case managers and evaluation professionals. In terms of importance there seems to exist social validity for the work ability evaluation as the evaluation results were considered representative of how clients function, and due to inclusive feedback from evaluation units. The official decision about sickness benefits was considered unrelated to the results from the previous work ability evaluation, lacking solid arguments and sometimes contradictory to other stakeholders’ recommendations which indicates poor social validity in terms of acceptability, comprehensibility and importance. Since the concept of social validity is new within a sickness insurance context, the contextual adaptations from one field to another may have led to some aspects explored in this study being extended parts of the original concept. This is a limitation in this study that should be further explored in order to facilitate the usage of social validity when evaluating work ability assessments.
